# *In-vitro* investigation of the remineralizing effect of sodium trimetaphosphate and fluoride varnishes using SEM-EDX and FTIR

**DOI:** 10.3389/fdmed.2026.1831227

**Published:** 2026-06-26

**Authors:** Imane El Ouarti, El Mostapha Lotfi, Abdelaziz Bouklouze, Faiza Abdallaoui

**Affiliations:** 1 Department of Conservative Dentistry and Endodontics, Faculty of Dental Medicine, Mohammed V University, Rabat, Morocco; 2 Laboratory of Spectroscopy, Molecular Modeling, Materials, Nanomaterials, Water and Environment, ENSAM, Mohammed V University, Rabat, Morocco; 3 Laboratory of Pharmacology and Toxicology, Faculty of Medicine and Pharmacy, Mohammed V University, Rabat, Morocco

**Keywords:** energy-dispersive x-ray spectroscopy (EDX), Fourier transform infrared spectroscopy, human enamel, remineralization, scanning electron microscopy, sodium trimetaphosphate

## Abstract

**Background:**

This study aimed to evaluate the remineralizing effect of sodium trimetaphosphate (STMP) combined with fluoride varnish on enamel subsurface lesions *in vitro*.

**Methods:**

Fifty human enamel samples were randomly assigned to five groups (*n* = 10): sound enamel (S), demineralized enamel (SL), 5% NaF, 2.5% STMP/NaF, and 5% STMP/NaF. Remineralization was performed using a pH-cycling model. Surface and subsurface changes were analyzed using SEM, EDX, and FTIR. Data were analyzed using ANOVA with Bonferroni *post hoc* tests (*p* < 0.05).

**Results:**

The 5% STMP/NaF group showed significantly higher fluoride content and Ca/P ratios compared to other groups. FTIR analysis demonstrated enhanced phosphate band intensity and reduced carbonate substitution.

**Conclusion:**

The addition of 5% STMP to fluoride varnish enhances enamel remineralization at both surface and subsurface levels.

## Background

Dental caries is a dynamic process alternating demineralization and remineralization phases (1). In enamel, this process occurs at the crystal surface and leads to dissolution of hydroxyapatite. The first clinical sign of enamel demineralization is represented by “white spot lesions” also referred to as an incipient or subsurface lesion (SL), consisting of a relatively intact surface layer overlying a demineralized sub-surface zone (2, 3). Prior to cavitation, therapeutic intervention using enamel remineralizing systems can arrest or reverse the carious process in enamel (4–6), thus preventing invasive surgical management of cavitated lesions and high economic cost of restorative procedures. While fluoride varnishes are the clinical gold standard for promoting remineralization, their efficacy is often limited to the outermost regions of the lesion, leaving deeper subsurface layers inadequately treated (7, 8).

Recent research suggests that Sodium Trimetaphosphate (STMP), a condensed inorganic phosphate (9), may synergize with fluoride to enhance mineral deposition in these deeper enamel regions (10, 11). However, the exact chemical mechanism by which STMP facilitates this process remains poorly understood. Most existing studies rely on polarized light microscopy or hardness testing, which lack the sensitivity to map elemental gradients or molecular structural changes within the enamel matrix (12–19). Despite the documented synergistic effects of STMP and fluoride, there is a lack of high-resolution chemical and molecular evidence regarding the mechanism of STMPmediated subsurface remineralization (17, 20–23). Specifically, the precise elemental distribution and mineral structural alterations occurring at depth remain uncharacterized (24).

Fourier Transform Infrared Spectroscopy (FTIR) is a highly efficient vibrational technique for analyzing biological hard tissues at the microscale. By identifying band positions and functional groups, FTIR tracks biochemical and structural changes in hydroxyapatite minerals during demineralization and remineralization (25–27). Complementing this, Energy Dispersive x-ray Spectroscopy (EDX) integrated with Scanning Electron Microscopy (SEM) provides a semi-quantitative assessment of enamel's elemental composition (28).

Given the analytical power of these techniques, this study aims to evaluate the *in vitro* effects of fluoride varnish supplemented with sodium trimetaphosphate (STMP) on the remineralization of human enamel caries-like lesions using both FTIR and EDX.

## Methods

### Selection of enamel blocks and ethical aspects

The present *in vitro* study was conducted on a sample of 50 extracted permanent human incisors and canines. These teeth were extracted for orthodontic reasons and were used after obtaining approval of the local ethical research committee (process Number 67/22). The number of teeth required in this study was calculated by using G.Power Software, then by comparing two means that were obtained from a preliminary study (with *α* = 0.05, *β* = 0.2, Power = 90%) (29, 30). The sample size required for the study was (10) enamel blocks per group.

Only teeth without caries, abrasions, erosions, stains, and restorations were included in the present study.

### Enamel blocks preparation

**Figure 1:**
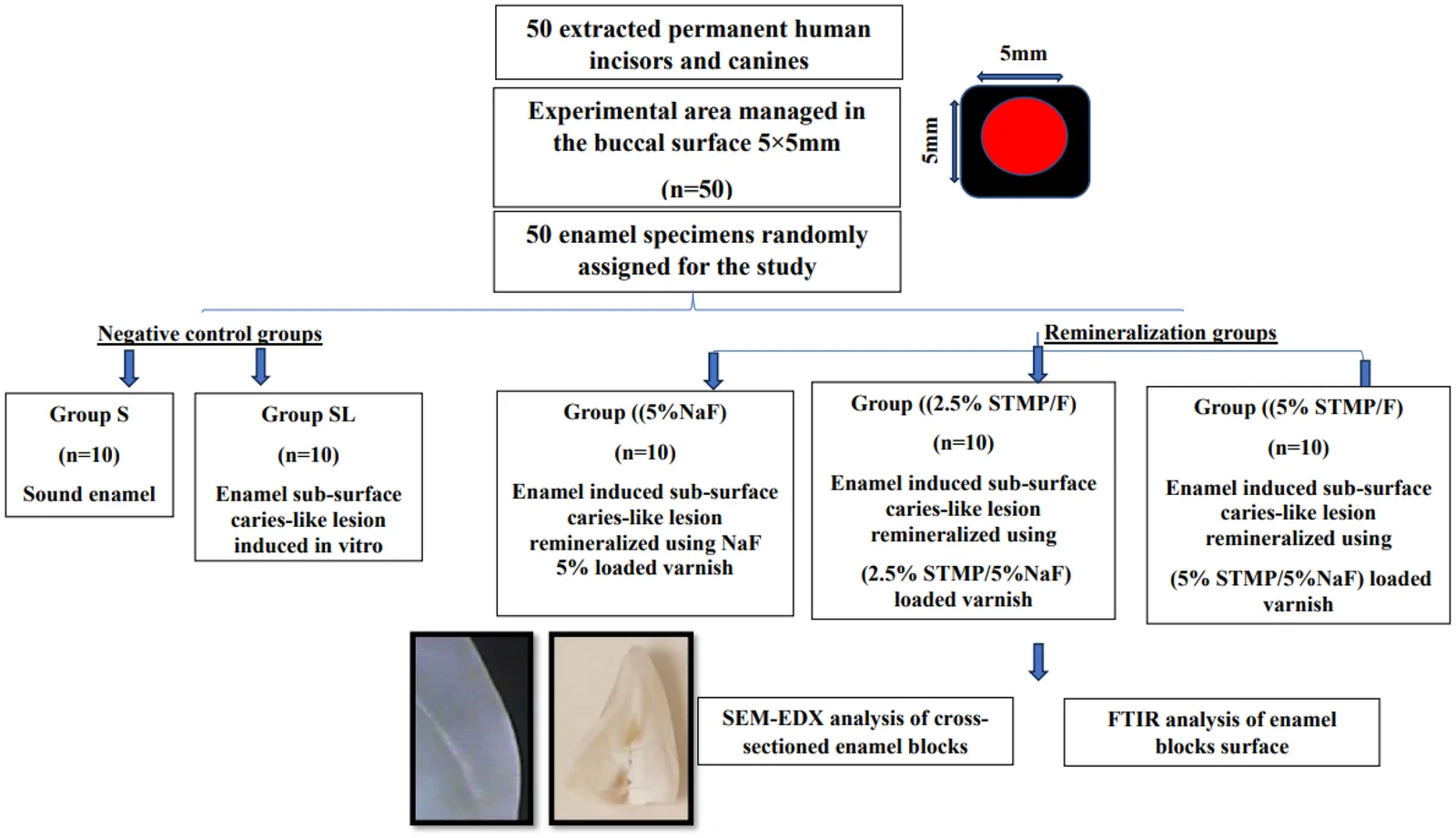
Flow chart of the study.

Enamel blocks were obtained from human permanent teeth previously stored in 2% formaldehyde solution pH = 7.0 for 30 days at room temperature (15, 31). The roots were cut with a diamond high speed saw with dental handpiece at the cemento-enamel junction. An experimental area of 5 mm × 5 mm was managed in the buccal surface of teeth to induce enamel subsurface lesions *in vitro* and remineralization of the induced carieslike lesions *in vitro*. Enamel blocks were coated with acid-resistant varnish on all sides except for the enamel buccal surface. The study design was summarized in (Figure 1).

### Varnishes formulation

The formulated varnishes contained 5% NaF with or without the addition of 5% STMP or 2.5% STMP. All varnishes were made up of the following components: artificial resin (35%), ethanol (12.5%), saccharin and deionized water. Fluoride at concentration of 5% was obtained from sodium fluoride (NaF, CAS 7681-49-4, Sigma-Aldrich, Germany), to which STMP (Na_3_O_9_P_3_, purity ≥95%, CAS 7785-84-4, Sigma-Aldrich, St.Louis, Mo, USA) was added either at concentrations of 2.5% or 5%. Fluoride concentrations in the varnishes were determined using an ion-specific electrode (9609 BN-Orion) and ion analyzer (Orion 720 A+) which was calibrated using standards at 2.0–32 µg F/mL, according to the protocol used by Manarelli et al. (12).

### Control and experimental treatment groups

Fifty enamel blocks were randomly assigned to 5 groups (*n* = 10) using a computergenerated randomization method and were submitted to one of the following treatments:

- The negative control group (S) of 10 sound and healthy enamel blocks without any natural or experimental demineralization.
- The negative control group (SL) of 10 enamel blocks submitted to a subsurface carieslike lesion induction protocol following a “demineralization” pH-cycling model.
- The experimental group (5%NaF) of 10 enamel blocks treated with NaF 5% loaded varnish and submitted to “remineralization” pH cycling model after caries-like lesion induction *in vitro*.
- The experimental group (2.5%STMP/F) of 10 enamel blocks treated with (5% NaF, 2.5% STMP) loaded varnish and submitted to “remineralization” pH cycling model after caries-like lesion induction *in vitro*.
- The experimental group (5%STMP/F) of 10 enamel blocks treated with (5% NaF, 5% STMP) loaded varnish and submitted to “remineralization” pH cycling model after caries-like lesion induction *in vitro*.

### Experimental groups treatment

#### Enamel subsurface lesion induction following a “demineralization” pH-cycling model (Group SL)

The pH-cycling regimen took 6 days, and the enamel blocks were kept at 37 °C for 6 h in 20 mL of the demineralizing solution (2.0 mmol/L calcium and phosphate in 75 mmol/L acetate buffer, pH 4.7; 0.04 μg Fluoride) and 18 h in 20 mL of the remineralizing solution (1.5 mmol/L Calcium, 0.9 mmol/L Phosphate, 150 mmol/L KCl in 0.1 mol/L cacodylic buffer, pH 7.0; 0.05 mg Fluoride) following a protocol modified from Queiroz et al. (32).

#### Treatment with varnishes and enamel subsurface lesion remineralization following a “remineralization” pH-cycling model

Enamel blocks featuring subsurface lesions-previously induced *in vitro* via the aforementioned demineralization pH-cycling model were subjected to a 6-day remineralization protocol (33). Initially, experimental varnishes were applied in a thin, uniform layer to the buccal surface of the specimens using a disposable microbrush. Approximately 10 µL of varnish was applied to each specimen. These groups included: 5% NaF (Group 5%NaF), 5%NaF plus 2.5% STMP (Group 2.5%STMP/F) and 5%NaF plus 5%STMP (Group 5%STMP/F). Following application, the specimens were incubated at 37 °C for 4 h in 20 mL of a remineralizing solution (1.5 mmol/L Calcium, 0.9 mmol/L Phosphate, 150 mmol/L KCl in 0.1 mol/L cacodylic buffer, pH 7.0; 0.05 mg Fluoride), followed by 2 h demineralization phase (2.0 mmol/L Calcium and Phosphate in 75 mmol/L acetate buffer, pH 4.7; 0.04 μg Fluoride).

The varnishes were subsequently removed using a blade and acetone, after which the blocks were transferred to fresh remineralizing solution for 18 h. this cycle was repeated daily for a total of 6 days.

### Scanning electron microscope/energy dispersive x-ray (SEM-EDX) analysis

After the experimental procedures, teeth were cut into two halves along the longitudinal axis in a bucco-lingual direction to produce cross sections of teeth by using high speed diamond saw (Isomet 2000, Buehler). The cross-sections of each enamel block were then embedded in self-curing acrylic resin with the cross-sectioned surfaces kept exposed. The sections were polished with water-cooled carburundom discs (320, 600, and 1,200 grit alumina papers; Buehler).

The sections were analyzed with an Environmental Scanning Electron Microscope thermoscientific Quattro S (Thermo Fisher Scientific Inc), with Ultra Dry detector operating at Low Vacuum, mode. Experimental conditions were set at the range of (2,500–35,000) ×magnification and 15 KV accelerating voltage. Elemental analysis was carried out with an energy dispersive x-ray analysis system (FEG-EDX) using a thermo-Noran (Thermo scientific, Rockford, IL, USA) NSS system7.

Line scans were collected along the line that goes from the resin into the enamel in order to determine elemental distribution in the cross-sectioned enamel surface. In each sample, 3 points were randomly selected from the enamel surface to the enamel depth and the mean values were calculated. Line scans through whole enamel cross-sectioned surfaces from the enamel surface to enamel depth were made at 50 points in scan with a dwell time per pixel of (5s) and 277 Total line scans, and high energy cut off (20 Kev) (30, 34).

### Fourier transform infrared spectroscopy (FTIR) analysis

The enamel surface of each specimen in the different studied groups was investigated with Fourier transform infrared spectroscopy (FTIR) without any further sample preparation. FTIR spectrometric investigations were performed with a Thermo Scientific™ Nicolet™ iS50 FTIR Spectrometer (Thermo Electron scientific Instruments LLC, Madison, WI USA) equipped with a Built-in, all reflective ATR unit (diamond crystal ATR).

On each sample, spectra were recorded from 4,000 to 450 cm^−1^ with a spectral resolution of 4 cm^−1^, each spectrum was the average of 32 scans. Each sample was scanned 3 times to check the reproducibility of the identical spectra. Raw IR spectra were converted in absorbance mode after gaining spectral data using OMNIC SPECTA software version 9.8 (Thermo Fisher Scientific Inc., Madison, WI USA), baseline correction and spectral normalization was performed using OMNIC Paradigm software (24, 25, 30).

The exact position of PO_4_^3−^ and CO_3_^2−^ was detected using Origin Pro software (Origin Pro 2023, origin Lab, Northampton, MA, USA).

### Statistical analysis

Quantitative data were described by means and standard deviations. Data were analyzed using One-Way ANOVA and Bonferroni correction for *post-hoc* comparisons. Data were analyzed using SPSS for windows software, version 21.0 (SPSS Inc. Chicago, IL, USA). All comparisons were performed at a significance level of *p* < 0.05.

## Results

### SEM enamel surface analysis

**Figure 2:**
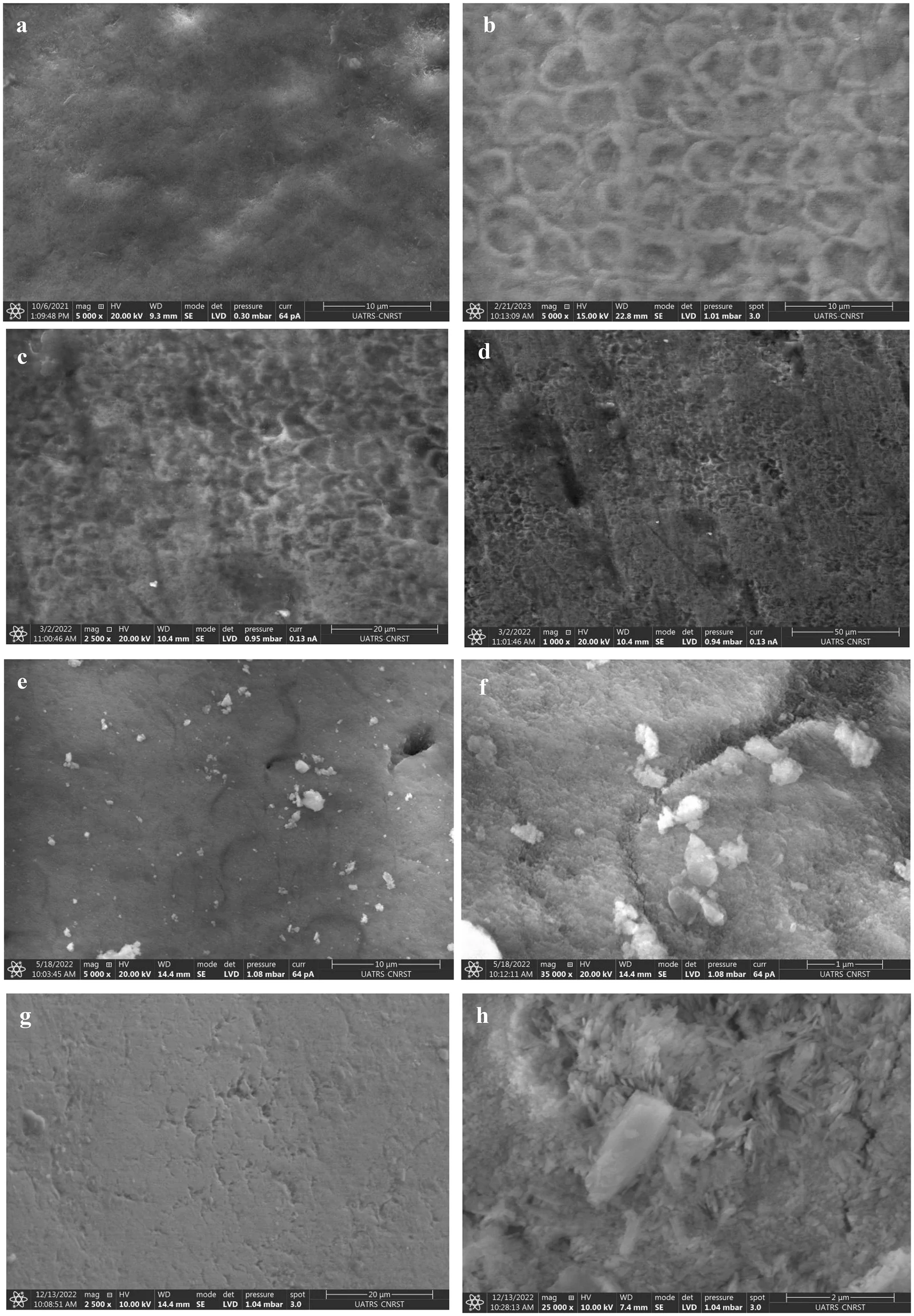
Representative scanning electron microscopic images of healthy enamel (Group S) **(a)**, enamel induced caries-like lesions (Group SL) **(b)**, enamel induced caries-like lesions remineralized with 5%NaF varnish (5%NaF) **(c,d)**, enamel induced caries-like lesions remineralized with (5%NaF, 2.5%STMP) experimental varnish (Group 2.5%STMP/F) **(e,f)**, enamel induced caries-like lesions remineralized with (5%NaF, 5%STMP) experimental varnish (5%STMP/F) **(g,h)**.

- Group S: healthy enamel showed unaltered enamel structure, no prismatic or interprismatic dissolution features have been detected under SEM magnification (Figure 2a).
- Group SL: enamel induced caries-like lesions *in vitro*, exhibited under SEM magnification enamel demineralization patterns. Marked interprismatic dissolution allowed identification of the enamel prismatic organization (Figure 2b).
- Group 5%NaF: enamel induced caries-like lesions remineralised with 5% NaF varnish showed reduced interprismatic spaces and higher mineral density in the periphery of the prisms than that seen in enamel induced caries like lesions (Group SL) (Figures 2c,d).
- Group 2.5%STMP/F: enamel induced caries-like lesions remineralised with (5% NaF, 2.5% STMP) experimental varnish showed under SEM magnification, reestablishment of enamel normal structure in some areas (Group S SEM images), interprismatic spaces were still detected in some areas (35.000 ×magnification) (Figures 2e,f).
- Group 5%STMP/F: enamel induced caries-like lesions remineralised with (5% NaF, 5% STMP) experimental varnish, showed under SEM magnification that interprismatic spaces were still detected in some enamel areas, but less wide than interprismatic spaces recorded in (Group R2). Mineral deposition in prismatic and interprismatic regions were identified in the form of needle-shape crystals (25.000 ×magnification) (Figures 2g,h).

### SEM-EDX analysis

Elemental analysis of the enamel was performed to determine the atomic percentages (At%) of Fluoride (F), Calcium (Ca), and phosphate (P). statistical analysis using One-way ANOVA revealed significant differences (*p* < 0.05) in elemental concentrations both at the surface (0 µm) and within the subsurface layers (25–150 µm) across all studied groups (Table 1).

**Table 1:** Means and standard deviation representations of the atomic percentage (At%) of investigated elements in enamel studied groups resulted from EDX analysis.

	S	SL	5%NaF	2.5%STMP/F	5%STMP/F	*p*
F At%						
0 µm	0.10 ± 0.02^a^	0.36 ± 0.10^b^	0.38 ± 0.01^b^	0.45 ± 0.08^c^	0.52 ± 0.04^c^	<0.001
25–100 µm	0.05 ± 0.01^a^	0.07 ± 0.01^b^	0.51 ± 0.03^c^	0.80 ± 0.04^d^	0.84 ± 0.05^d^	<0.001
100–200 µm	0.75 ± 0.04^a^	0.51 ± 0.03^b^	0.54 ± 0.03^b^	0.51 ± 0.06^b^	0.58 ± 0.03^c^	<0.001
Ca At%						
0 µm	16.25 ± 1.54^a^	24.15 ± 5.12^b^	15.71 ± 7.13^c^	28.74 ± 12.04^d^	30.76 ± 19.87^d^	<0.05
25–100 µm	19.79 ± 1.08^a^	18.06 ± 2.98^b^	18.43 ± 2.66^c^	18.98 ± 5.40^c^	20.24 ± 4.83^d^	<0.001
100–200 µm	19.30 ± 0.62^a^	20.08 ± 3.16^b^	19.78 ± 1.42^b^	20.10 ± 6.80^c^	20.90 ± 4.76^c^	<0.001
P At%						
0 µm	8.97 ± 0.38^a^	15.18 ± 3.69^b^	11.64 ± 1.38^b^	20 ± 9.85^c^	18.29 ± 9.38^d^	<0.001
25–100 µm	11.80 ± 0.18^a^	12.02 ± 1.20^b^	12.05 ± 0.81^b^	12.65 ± 3.32^c^	12.74 ± 2.28^c^	<0.001
100–200 µm	13.07 ± 0.14^a^	14.58 ± 1.34^b^	12.82 ± 0.83^c^	11.83 ± 4.50^b^	14.37 ± 1.01^b^	<0.001
Ca/P ratio						
0 µm	1.65 ± 0.10^a^	1.59 ± 0.11^b^	1.62 ± 0.12^b^	1.50 ± 0.14^c^	1.83 ± 0.12^d^	<0.001
25–100 µm	1.55 ± 0.12^a^	1.47 ± 0.06^b^	1.58 ± 0.10^c^	1.57 ± 0.18^c^	1.73 ± 0.14^d^	<0.05
100–200 µm	1.55 ± 0.01^a^	1.53 ± 0.16^a^	1.58 ± 0.07^c^	1.60 ± 0.03^c^	1.58 ± 0.07^c^	<0.05

Means followed by different letters (lowercase in rows) are different.

Specifically, the 5%STMP/NaF group exhibited significantly higher levels of F and Ca compared to all other experimental groups. Regarding Phosphate, the 2.5% and 5%STMP/F groups demonstrated the highest At% values at the surface and throughout the deep enamel layers (25–100 µm).

Furthermore, the 5% STMP/F group demonstrated significantly higher Ca/P ratios than the sound enamel control (Group S).

**Figure 3:**
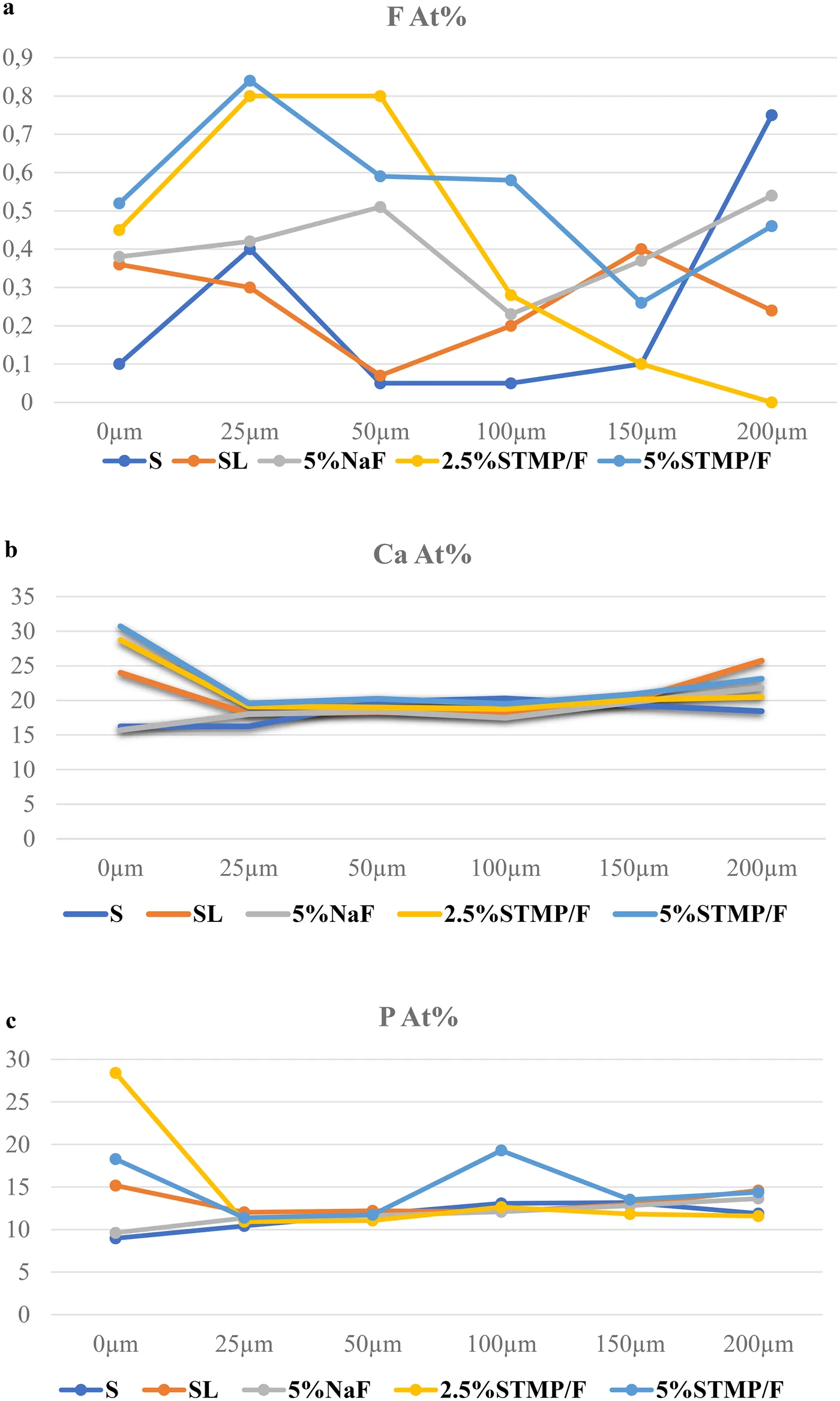
Representation of the mean atomic percentage (At%) variation of fluoride (F) **(a)**, calcium (Ca) **(b)**, phosphate (P) **(c)** at the enamel surface and in the enamel depth in the studied groups.

The spatial distribution and fluctuations of F, Ca, and P from the surface to the deep enamel layers are illustrated in (Figure 3).

### FTIR analysis

**Figure 4:**
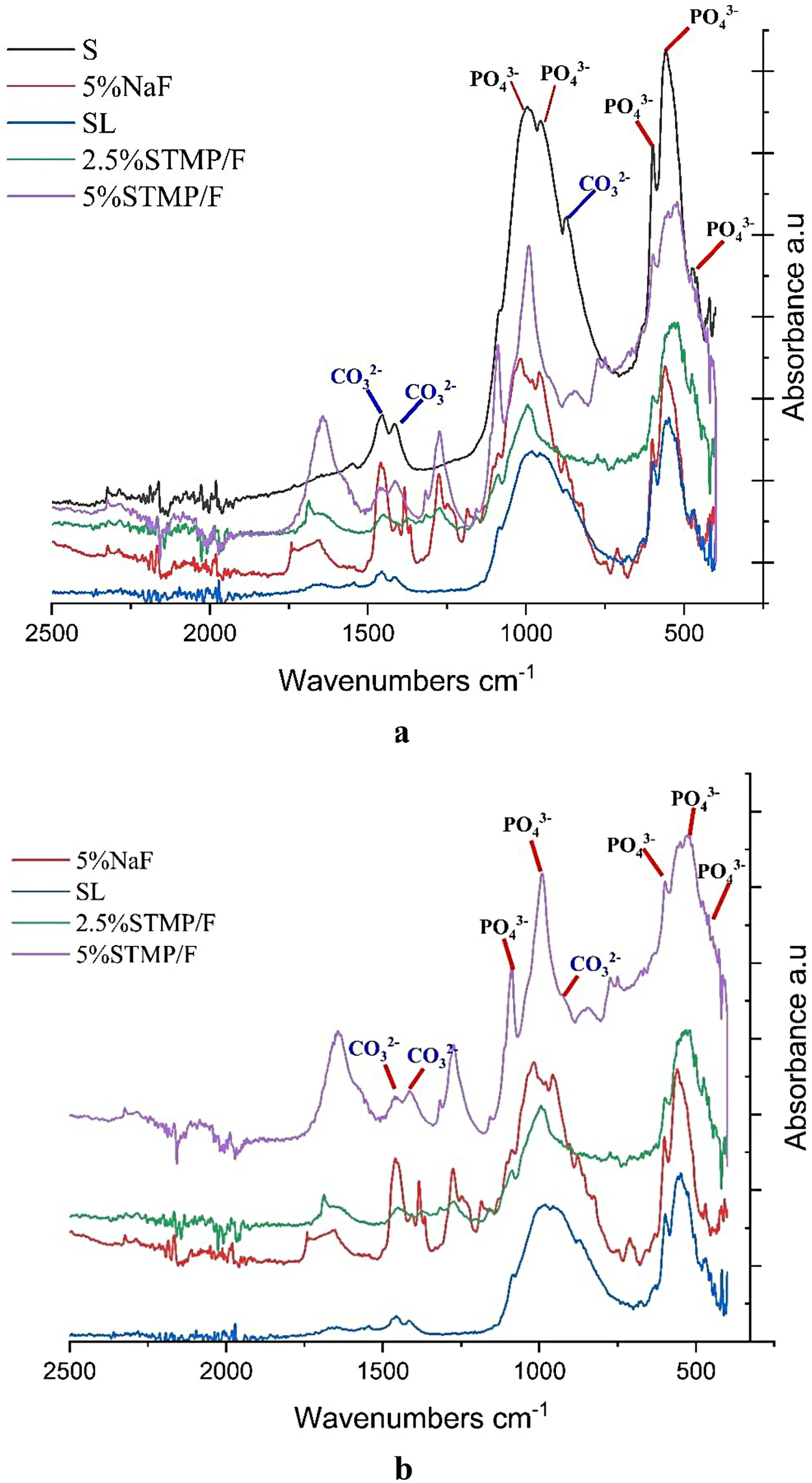
**(a,b)** Superposition of FTIR spectra of Group S, Group SL, Group 5%NaF, Group 2.5%STMP/F, Group 5%STMP/F.

Infrared spectroscopy was employed to investigate structural modifications within the demineralized and remineralized enamel across the experimental groups. FTIR analysis of the enamel surface enabled the detection of carbonate CO_3_^2−^ and phosphate PO_4_^3−^ spectral peaks relative to characteristic apatite bands. Variations in the infrared spectra, specifically in peak intensities, indicated structural alterations to the stoichiometric apatite (Figures 4a,b).

Characteristic bands for PO_4_^3−^ groups in the enamel were observed at 962 cm^−1^(*v*_1_), 470 cm−1(*v*_2_), 568–602 cm−1 (*v*_4_), and 1,015 cm^−1^ (*v*_3_). Similarly, characteristic bands for CO_3_^2−^ groups were identified at 875 cm^−1^(*v*_2_), and at 1,415, 1,460cm^−1^ (*v*_3_) (35, 36).

The phosphate vibrational modes *v*_1_ at 962 cm^−^, *v*_3_ at 1,015 cm^−1^ and *v*_4_ at 568–602 cm^−1^ exhibited distinct intensity shifts across the SL, 2.5% STMP/F, and 5% NaF groups. Notably, the 5% STMP/F group consistently demonstrated the highest peak intensities for all PO_4_ modes.

Second-derivative analysis revealed peaks at approximately 570 cm^−1^for both STMP/F groups, suggesting an evolution of the phosphate environment corresponding with crystal maturation. Furthermore, additional second-derivative peaks identified at 1,087 cm^−1^ and 1,090 cm^−1^ for the 5% and 2.5% STMP/F groups, respectively, likely reflect non-stoichiometry or the incorporation of acid phosphate-containing species.

Structural changes were also evidenced by a shift in the *v*_3_PO_4_ (1,015 cm^−1^) peak toward lower wavenumbers in the STMP/F groups. This red shift indicates an increase in the P–O bond length, necessitating a compensatory shortening and subsequent strengthening of the adjacent Ca–O bonds.

Regarding carbonation, intensity shifts in the *v*_2_CO_3_ (875 cm^−1^) and *v*_3_CO_3_ (1,415, 1,460 cm^−1^) peaks were observed in both STMP/F groups. In contrast, the 5% NaF group exhibited more pronounced carbonate bands, suggesting enhanced carbonate-for-phosphate substitution within the superficial enamel. Conversely, Group SL demonstrated a severe depletion of both PO_4_^3−^ and CO_3_^2−^ signals when compared to Group S.

**Figure 5:**
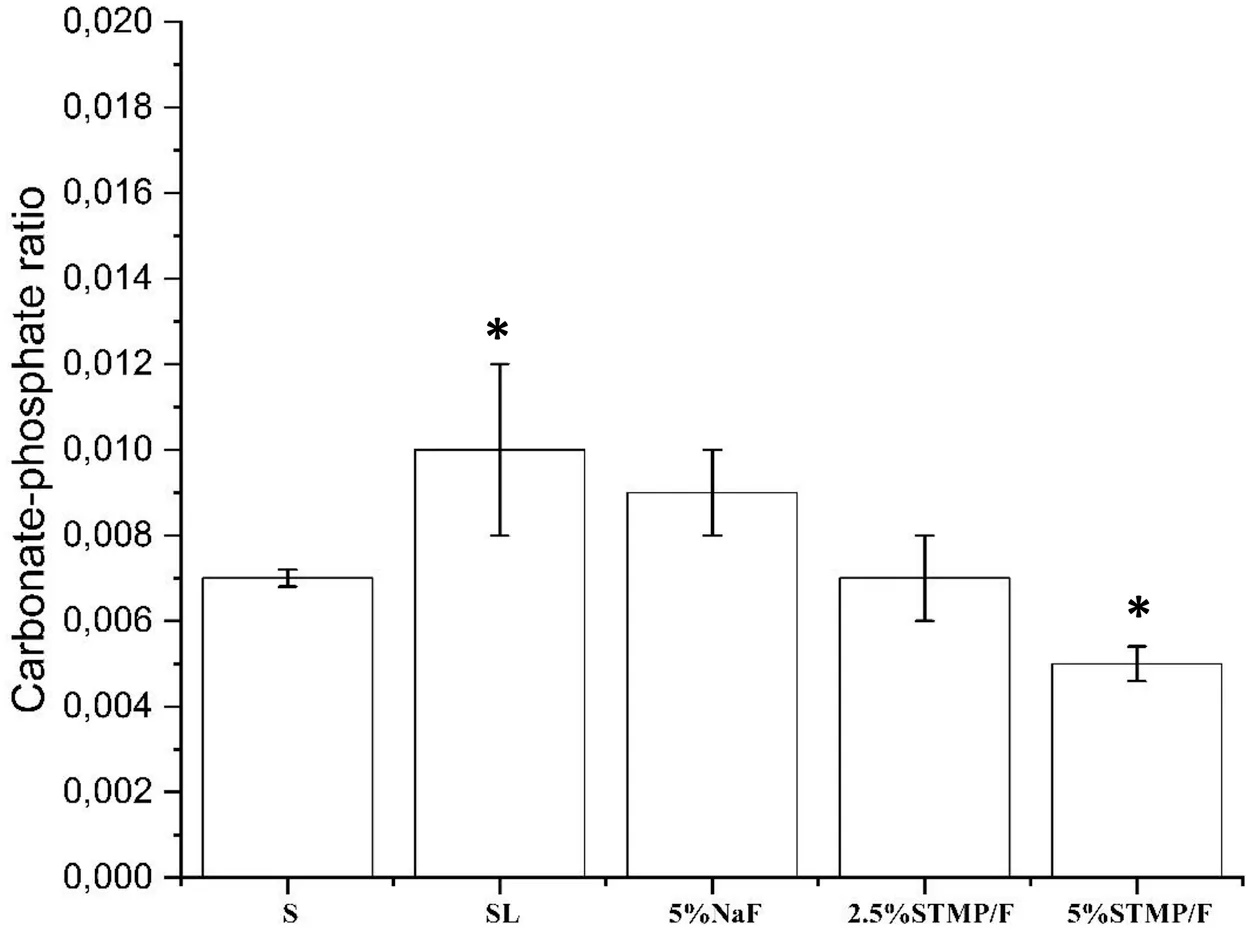
The carbonate to phosphate ratios calculated from infrared spectra for Group S, Group SL, Group 5%NaF, Group 2.5%STMP/F, and Group 5%STMP/F. Data represented as mean ± SD. *Statistically significant at *p* < 0.05.

The carbonate-to-phosphate ratio was determined from the FTIR spectra by calculating the ratio of the integrated area of the carbonate *v*_2_ band (850–890 cm^−1^) to that of the phosphate *v*_1_ and *v*_3_ (900–1,200 cm^−1)^ bands. Data are presented as mean ± SD (Figure 5). The carbonate-to-phosphate ratio was significantly higher in Group SL compared to all other experimental groups (ANOVA, *p* < 0.001). Conversely, Group 5% STMP/F exhibited the lowest carbonate-to-phosphate ratio among all studied groups (ANOVA, *p* < 0.05).

**Figure 6:**
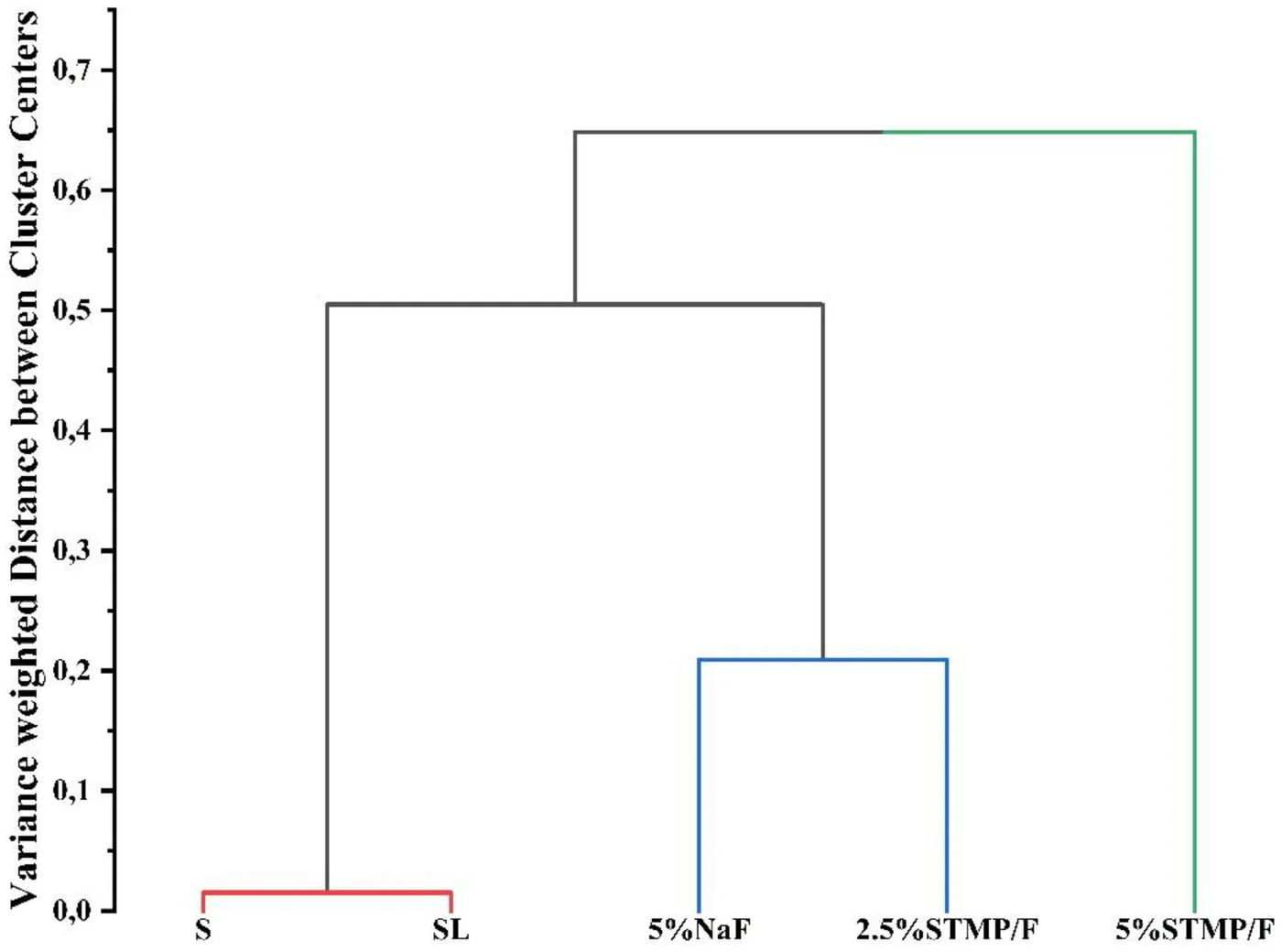
Hierarchical cluster analysis (HCA) for FTIR spectra in the spectral range of 470–2,500 cm^−1^. S, healthy enamel; SL, enamel induced subsurface caries-like lesions; 5%NaF, enamel induced caries-like lesions remineralized with 5%NaF varnish; 2.5%STMP/F, enamel induced caries-like lesions remineralized with 5%NaF, 2.5%STMP experimental varnish; 5%STMP/F, enamel induced caries-like lesions remineralized with 5%NaF, 5%STMP experimental varnish.

The average FTIR spectra of the experimental enamel groups were subjected to multivariate hierarchical cluster analysis (HCA) using the PLS Toolbox software (v8.2.1, Eigenvector Research Inc., USA) operating within the MATLAB environment (R2018b, The MathWorks, USA). Sample similarities were calculated using Ward's algorithm across the spectral range of 470–2,500 cm^−1^. The resulting dendrogram (Figure 6) illustrates the clusters obtained, where the vertical axis represents the dissimilarity between clusters and the horizontal axis represents the variables and groups. HCA demonstrated greater spectral heterogeneity in the 5% STMP/F group compared to the other experimental groups.

## Discussion

The present study demonstrates that the incorporation of 5% Sodium Trimetaphosphate (STMP) and 5% NaF into a varnish formulation results in significantly higher enamel remineralization compared to both 2.5% STMP/5% NaF and 5% NaF alone. This enhanced remineralization was confirmed via SEM-EDX and FTIR analysis, which revealed superior mineral deposition. These findings align with previous literature suggesting that STMP, when synergistically combined with fluoride (F), facilitates an increased flux of calcium and phosphate ions, thereby optimizing the remineralization process (19, 37). Notably, this formulation appears capable of promoting deep subsurface remineralization, addressing a key limitation of conventional fluoride treatments, which are typically restricted to the outermost layers of carious lesions (38, 39).

### Analysis of remineralization effects

In the present study, enamel samples with induced subsurface lesions exhibited significantly higher atomic percentages (At%) of Fluoride (F), Calcium (Ca), and Phosphate (P) following remineralization with the 5% STMP/5% NaF varnish compared to the control and other experimental groups. While stoichiometric hydroxyapatite is characterized by a Ca/P ratio of approximately 1.67 (40), the 5% STMP/5% NaF group yielded significantly higher ratios both at the enamel surface (1.83 ± 0.12) and within the lesion body (25–100 µm; 1.73 ± 0.14).

These findings suggest that the 5%STMP/5%NaF formulation enhances both phosphate adsorption and calcium diffusion throughout the enamel surface and into the deeper lesion layers. In contrast, the 2.5% STMP/5% NaF formulation failed to achieve this dual effect, primarily promoting superficial phosphate adsorption without facilitating significant calcium diffusion at depth. Consequently, it is proposed that a specific STMP/F molar ratio is requisite to optimize the concurrent ionic diffusion of Ca and P during the remineralization of carious lesions. These findings are consistent with previous reports in the literature (16–18, 21, 41).

Enamel subsurface lesions that received no remineralization treatment (Group SL) exhibited the lowest Ca/P ratios, with values of 1.59 ± 0.11 at the surface and 1.47 ± 0.06 at a depth of 25–100 µm. This mineral deficit is attributable to the substantial leaching of Ca and P ions during the demineralization phase. Consequently, these results validate the efficacy of the *in vitro* pH-cycling model employed in this study for the induction of standardized subsurface lesions.

Furthermore, the data indicate that a 5% STMP concentration exerts a superior remineralization effect at the chemical level compared to a 2.5% concentration, as evidenced by SEM-EDX and FTIR analysis. This dose-dependent efficacy corroborates the findings of Paiva et al. (42). The mechanism underlying the STMP-mediated remineralization may be explained by the cross-linking between Ca ions and the trimetaphosphate (TMP) cyclic structure. This interaction, facilitated by the binding sites of the phosphate groups PO_4_^3−^, promotes the formation of neutral species, such as hydrogen fluoride (HF^°^) and calcium phosphate (CaHPO_4_^°^) (12, 43, 44). These neutral complexes possess a higher diffusion coefficient than ionic calcium, thereby facilitating deeper penetration and mineral deposition within the lesion body (45).

At 5%, STMP formulated varnish did not interfere with fluoride adsorption in enamel surface or deep layers (25–150 µm), contradicting previous reports suggesting concentrations above 3% might reduce the synergistic anticaries effect between NaF and STMP (46). This divergence is governed by distinct vehicle kinetics, substrate morphologies, and analytical sensitivities. Most of the previous studies evaluated shortterm toothpaste slurry on highly porous bovine enamel using surface hardness measurements which detect physical indentations resistance but lack chemical specifity (39, 46). These differences may be explained by the prolonged contact matrix of the varnish, paired with a high fluoride payload, prevents the competitive surface clogging often seen in transient bovine models. Instead, it facilitates a deeper, synergistic ion diffusion into the less porous human tissue. This structural and elemental optimization is directly confirmed by FTIR and SEM-EDX data in the present study, which reveal high-quality apatitic lattice reorganization and dense mineral deposition that macroscopic hardness metrics alone cannot characterize.

While recent studies highlight 5% STMP/F varnishes as highly effective against erosive wear in enamel, further elemental analysis is required to determine if high STMP concentrations compete with fluoride adsorption (42, 47).

In Group SL, alterations in PO_4_^3−^ bands indicate structural changes to stoïchiometric apatite following subsurface lesion induction. For the 5%NaF Group, a second peak near 1,015 cm^−1^ suggests altered atomic bonding in superficial enamel, implying that NaF varnish alone fails to fully reestablish the hydroxyapatite structure (20). Furthermore, increase *v*_3_CO_3_ band intensity indicates carbonate substitutions that weaken the enamel lattice (24).

### STMP/F synergistic effects

The 5% STMPS/F Group demonstrated superior remineralization patterns characterized by:

- Enhanced bonding: second derivative peaks at ∼570 cm^−1^ and *v*_3_PO_4_ shifts suggest P-O bond lengthening and Ca-O bond strengthening as the crystal matures (20, 36, 48).
- Mineral density: elemental analysis confirmed significantly higher calcium (At%) in this group. The combination promotes calcium adsorption and deep diffusion, supported by EDX findings.
- Structural integrity: A significantly lower Carbonate/phosphate ratio favors fluoride and phosphate substitutions over carbonate, strengthening the enamel composition.
- Microscopic Morphology: SEM imaging revealed needle-shaped mineral deposits in both prismatic and interprismatic regions; showing improved space reestablishment compared to 5% NaF or 2.5% STMP/F.

FTIR Hierarchical Cluster Analysis (HCA) showed the highest heterogeneity in the 5% STMP/F Group, reflecting a complex and effective redistribution of minerals. Ultimately, the 5% STMP/5%NaF combinations facilitates superior crystallographic repair and mineral diffusion through enlarged interprismatic spaces in subsurface lesions.

### Mechanism significance

The present study findings suggest that, STMP acts as a delivery enhancer, it binds to Ca^2+^ and Ca F ions, facilitating the diffusion of minerals into the lesion's depth. This overcomes a common limitation of fluoride-only treatments, which typically only remineralize the enamel surface (11, 49, 50). By using human enamel rather than bovine tissue, this study provides a more accurate representation of clinical effects, as it accounts for specific human ultrastructural characteristics (51, 52).

These findings suggest potential applications in preventive dentistry; however, clinical studies are required. The present findings may support the development of enhanced fluoride varnish formulations targeting early enamel lesions and white spot lesion management.

In alignment with bioactive materials engineered to release calcium, phosphate, and fluoride ions in acidic environments, the STMP/ F varnish holds promise for caries prevention, especially among high-risk pediatric and elderly patients. Nonetheless, further studies evaluating its performance under fluctuating pH and temperature conditions remain necessary (53, 54).

### Study limitations

While results are promising, the study's scope was limited by:

- The absence of micro-CT or depth-microhardness testing to quantify the exact depth of remineralization.
- The lack of longitudinal data to assess long-term stability.
- The *in vitro* nature of the study, which requires further *in vivo* validation to account for the complexity of the oral environment.

## Conclusion

Within the limitations of this *in vitro* study, fluoride varnish supplemented with 5% STMP significantly enhanced both surface and subsurface enamel remineralization compared to fluoride alone. Further *in vivo* studies are required to validate these findings.

## Data Availability

The original contributions presented in the study are included in the article/Supplementary Material, further inquiries can be directed to the corresponding author.
